# Salivary β‐amyloid index 42/40 as a predictor in the diagnosis of Alzheimer's disease

**DOI:** 10.1002/alz70856_106468

**Published:** 2026-01-09

**Authors:** Gustavo A A Santos

**Affiliations:** ^1^ USP ‐ University of Sao Paulo, Ribeirao Preto, Brazil; Sao Leopoldo Mandic Araras School of Medicine, Araras, Brazil; UNICAMP, Campinas, Brazil

## Abstract

**Background:**

Recent laboratory analysis techniques have made it possible to use saliva as a fluid capable of being used in the search for biomarkers that may be correlated with Alzheimer's disease. In this sense, our group evaluated the salivary concentrations of the beta amyloid peptide in the 40 and 42 amino acid fractions and, after observing the feasibility of the analysis, correlated the values found.

**Method:**

This is a case‐control study conducted in patients diagnosed with probable Alzheimer's disease (AD) and cognitively healthy patients without AD. The study was conducted in Ribeirão Preto, SP, Brazil. A total of 76 participants were invited to participate in this experiment, as follows:

• Elderly group without AD: 26 cognitively healthy elderly individuals without a diagnosis of AD, aged 65 years or older;

• Adults without AD group: 25 cognitively healthy non‐elderly adults without a diagnosis of AD, aged 19 to 59 years;

• Elderly group with AD: 25 patients with a diagnosis of probable AD, aged 65 years or older.

We used the ELISA method after very specific treatment of saliva, to the point of making it stable and free of interferents so that the analyses could be performed. Approval by the Research Ethics Committee, Opinion Number: 3.130.636

**Result:**

The calculation of the numbers corresponding to the salivary concentrations of Abeta 42/40 in men and women was performed, in addition to the general average and the results did not indicate consistency regarding the values when compared to the literature, to indicate a probable diagnosis of AD.

**Conclusion:**

Although the results of salivary Abeta42 may indicate the viability of using this biomarker, when applying the Abeta42/40 ratio, the results were not consistent with the clinical characteristics of patients with AD.

